# Development of a Mucosal Immune-Enhancing Oral Vaccine Candidate Against Porcine Epidemic Diarrhea Virus Using *Lactobacillus paracasei*

**DOI:** 10.3390/ani16030471

**Published:** 2026-02-03

**Authors:** Yijie Yang, Ling Sui, Yuliang Zhao, Jiaxuan Li, Fengsai Li, Wen Cui, Yanping Jiang, Lijie Tang, Dianzhong Zheng, Xiaona Wang

**Affiliations:** 1 College of Veterinary Medicine, Northeast Agricultural University, Harbin 150030, China; s230601082@neau.edu.cn (Y.Y.); b210601009@neau.edu.cn (L.S.); zylianges@163.com (Y.Z.); lijiaxuan.1993@163.com (J.L.); cuiwen_200@163.com (W.C.); jiangyanping8198@163.com (Y.J.); tanglijie@163.com (L.T.); 2 Heilongjiang Key Laboratory for Animal Disease Control and Pharmaceutical Development, Harbin 150030, China; 3 Hebei Key Laboratory of Preventive Veterinary Medicine, College of Animal Science and Technology, Hebei Normal University of Science and Technology, Qinhuangdao 066004, China; 4 Institute of Animal Husbandry, Heilongjiang Academy of Agricultural Sciences, Harbin 150030, China

**Keywords:** *Lactobacillus paracasei*, PEDV S1 protein, dendritic cell-targeting peptide, M cell-targeting peptide, LTB, oral vaccine

## Abstract

Porcine epidemic diarrhea virus (PEDV) is a highly infectious virus that leads to severe diarrhea and high death rates in neonatal piglets. Because PEDV primarily infects the intestinal mucosa, the induction of effective mucosal immunity through oral vaccination represents a promising strategy for disease prevention. In this study, a recombinant *Lactobacillus paracasei* (*L. paracasei*) strain expressing a multicomponent fusion antigen composed of the PEDV S1 protein, M cell- and dendritic cell-targeting peptides, and the mucosal adjuvant LTB was constructed as a candidate oral vaccine. Pregnant mice orally immunized with the recombinant strain exhibited significantly increased levels of PEDV-specific serum IgG as well as secretory IgA (SIgA) in intestinal mucus and feces, both of which showed in vitro neutralizing activity. In addition, oral immunization markedly enhanced cellular immune responses, as indicated by elevated serum levels of IFN-γ, IL-2, IL-4, and IL-10. Notably, newborn mice delivered by immunized dams displayed significantly higher levels of PEDV-specific SIgA, demonstrating effective maternal antibody transfer. These results indicate that the recombinant *L. paracasei* strain can robustly induce humoral, cellular, and mucosal immune responses and confer maternal immune protection. This study emphasizes the possibility of oral vaccinations based on *L. paracasei* as a viable approach to the prevention and management of epidemic diarrhea in piglets.

## Simple Summary

Porcine epidemic diarrhea virus (PEDV) is a highly contagious pathogen that infects the intestinal tract of piglets, causing severe diarrhea and high newborn piglet deaths, causing the swine industry to suffer large financial losses. Given that PEDV primarily targets the intestinal mucosa, oral vaccines designed to stimulate mucosal immunity may offer effective protection. In this study, we developed a recombinant strain of *Lactobacillus paracasei* that expresses a fusion antigen comprising the PEDV S1 protein, immune cell-targeting peptides, and a mucosal adjuvant. Oral immunization of pregnant mice with this recombinant strain resulted in a significant increase in PEDV-specific antibodies in both the bloodstream and intestinal tract, with these antibodies demonstrating the ability to neutralize the virus in vitro. Furthermore, the vaccine enhanced cellular immune responses, evidenced by elevated levels of immune-related cytokines. Notably, newborn mice born to immunized mothers exhibited higher levels of PEDV-specific antibodies, indicating effective transfer of maternal immunity. These findings illustrate that the recombinant *L. paracasei* oral vaccine can elicit robust mucosal, humoral, and cellular immune responses while providing maternal immune protection. Porcine epidemic diarrhea can be prevented and controlled with this strategy, which is both practical and promising.

## 1. Introduction

Porcine epidemic diarrhea (PED) is an acute enteroviral disease caused by the porcine epidemic diarrhea virus (PEDV) [1,2]. Clinical symptoms include severe diarrhea, vomiting, and fluid loss, and the sickness manifests abruptly and progresses rapidly. PEDV is capable of infecting pigs across all age groups; however, infected suckling piglets may experience mortality rates as high as 100% [3]. The appearance of highly pathogenic mutant strains in China in 2010 has resulted in a reduction in the protective efficacy previously associated with conventional inactivated and attenuated vaccines [4]. The spike (S) protein of PEDV is also known as the viral fibrillar protein [5]. The structure includes two smaller segments, S1 and S2, where the S1 subunit is vital for initiating the formation of immunogenic neutralizing antibodies [6,7]. Consequently, researchers often utilize the S1 subunit from the immunodominant region of the S protein as an immunogen [8]. Considering that porcine intestinal cells are the principal targets of PEDV infection, direct stimulation of the gastrointestinal tract can elicit mucosal immunity, thereby effectively inhibiting viral replication. SIgA, produced by the mucosal surfaces, plays a crucial role in defending against PEDV infection [9,10]. Therefore, lactic acid bacteria-based oral vaccines that can stimulate mucosal immunity and generate protective mucosal antibodies are an effective approach for preventing PED.

Lactic acid bacteria (LAB), especially *Lactobacillus*, can colonize the gastrointestinal tract, thereby simulating the natural infection process and directly stimulating the intestinal mucosa to elicit an immune response [11,12]. In addition, *Lactobacillus* have the characteristics of being safe, and are widely used as carriers for vaccines [13]. However, the low efficiency of antigen presentation has greatly limited its application [14]. Therefore, the new type of LAB-based oral vaccine with mucosal adjuvants or mucosal targeting, which is capable of enhancing mucosal immunity, represents a new direction for combating mucosal infections.

Dendritic cells (DCs) are essential in controlling immune responses driven by T cells and B cells. They have the ability to identify, capture, digest, and deliver antigens to T cells. Consequently, DCs are frequently targeted for antigen delivery [15]. Specialized immune cells known as microfold cells (M cells) are essential for the monitoring and phagocytosis of foreign substances such as pathogens, microbes, and antigens. These cells are integral to antigen transport, facilitating the translocation of diverse foreign substances to lymphoid tissues located within the intestinal mucosa. Recent research has shown that incorporating dendritic cell-targeting and M cell-targeting peptides into oral *Lactobacillus* delivery systems markedly improves the efficiency of antigen recognition and uptake [16,17], which ultimately results in a more robust immune response [18,19]. The enterotoxin-producing bacteria *Escherichia coli* secretes a substance called heat-labile enterotoxin (LT), which is composed of two subunits: A and B [20]. The non-toxic B subunit (LTB) exhibits significant immunomodulatory properties and is capable of directly binding to receptors on intestinal mucosal cells, thereby contributing to the mucosal immune response. LTB has the capacity to enhance immune responses, when utilized as a mucosal immune adjuvant in conjunction with other antigens [21]. The immunomodulatory properties of LTB enable the regulation of immune responses through multiple pathways, thereby enhancing activation. It facilitates the entry of exogenous antigens into the cytoplasm and their binding to MHC molecules, which augments antigen presentation [22]. Additionally, LTB functions as an adjuvant, synergizing with antigens to promote specific antibody production. It also promotes the maturation and activation of DCs, stimulates T cell signal expression on antigen-presenting cells to initiate lymphocyte differentiation, and increases the expression of activation markers on B lymphocytes. Furthermore, LTB enhances the secretion of cytokines such as TNF-α, IL-6, and IL-10, induces Th1/Th2 cytokine responses against conjugated antigens, and maintains immune homeostasis by inducing apoptosis in CD8^+^ T cells, thus comprehensively regulating both innate and adaptive immunity [23,24,25].

In this study, *Lactobacillus* of porcine was applied to express the S1 antigen that protects against PEDV. To enhance mucosal-specific immunity, the development of mucosal immune-enhancing oral vaccine strains against PEDV involved the integration of the M cell-targeting peptide Co1, the DCs-targeting peptide 6aa, and the mucosal immune adjuvant LTB. Subsequently, the immunological responses elicited by the vaccine candidate strain were assessed through oral administration to pregnant mice. By evaluating the immunological effects of the vaccine candidate, this approach aimed to offer both theoretical and practical foundations for the evolution of innovative, safe, and effective vaccines against PEDV.

## 2. Materials and Methods

### 2.1. Bacterial Strain, Virus, and Plasmid

**Figure 1:**
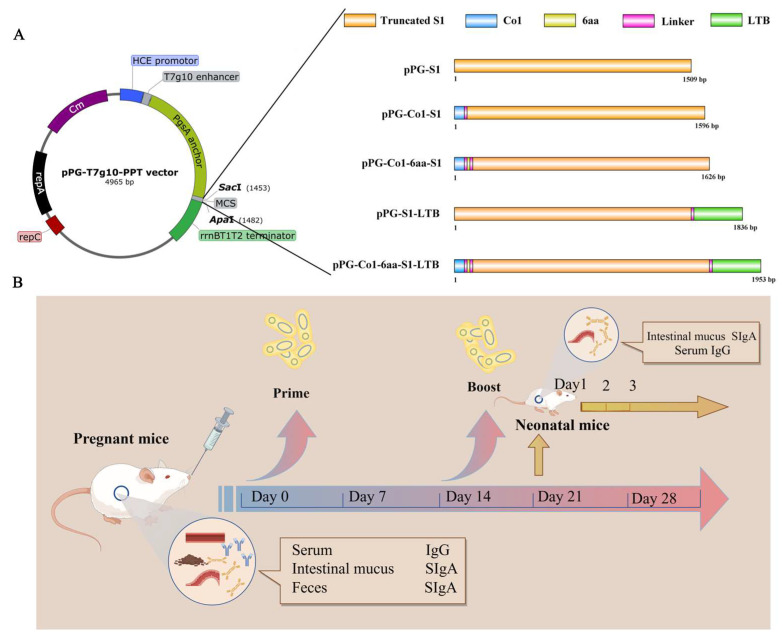
Schematic diagram of recombinant *Lactobacillus* construction and maternal immunization experiment. (**A**) Illustrative diagram showing the creation of recombinant plasmids. HCE strong constitutive promoter; T7g10 transcriptional enhancer; PgsA anchor from Bacillus subtilis; COE coding sequence; DC-targeting peptide; M cell-targeting peptide; rrnBT1T2 terminator; replicon C (rep C); replicon A (rep A); chloramphenicol coding sequence (Cm^+^). (**B**) Immunization protocol and sampling schedule. Mouse groups were given oral immunizations with recombinant *Lactobacillus* or PBS on days 1, 2, and 3. A booster dose was administered on days 15, 16, and 17, with samples subsequently collected at various intervals post-vaccination.

The growth of *Lactobacillus paracasei 27-2* (*L. paracasei 27-2*) occurred in de Man Rogosa and Sharpe (MRS) broth at 37 °C. PEDV strain [26] (GenBank: MW561264.1) was kept in our laboratory after being isolated from the intestines of pigs in Heilongjiang, China. The constitutive expression plasmid pPG-T7g10-PPT (as shown in Figure 1A) containing a HCE strong constitutive promoter cloned from the d-amino acid aminotransferase, a T7g10 transcriptional enhancer, a PgsA anchor from Bacillus subtilis for surface-displaying heterologous protein on the cell membrane, and an rrnBT1T2 terminator was constructed by our laboratory [27,28]. The recombinant *Lactobacillus* pPG-S1/*27-2* [29], recombinant *Lactobacillus* pPG-6aa-S1/*27-2* and pET28a-LTB/*TG1*, were developed in our laboratory.

### 2.2. Development of Recombinant Porcine-Derived L. paracasei 27-2

The construction process of the recombinant expression vector is depicted schematically in Figure 1. The *Co1-S1* fragment was PCR-amplified from the recombinant plasmid pMD19Ts-S1 using primer pair Co1-F and Co1-R, and subsequently, the plasmid pPG-PPT was modified to include the *Co1-S1* fusion gene, resulting in the recombinant plasmid pPG-Co1-S1. The pPG-Co1-6aa-S1 was constructed as above, and the primer pairs used were Co1-F and Co1-R. Additionally, primer pairs S1-F1/S1-R1 and LTB-F/LTB-R were employed to amplify the *S1* and *LTB* genes from the pMD19Ts-S1 and pET28a-LTB, respectively. Subsequently, the two sequences underwent fusion PCR utilizing primer S1-F1 and LTB-R, and the *S1-LTB* fragment was ultimately cloned into the pPG-PPT. The pPG-S1-LTB was amplified with primers C6-F and LTB-R to generate the Co1-6aa-S1-LTB fragment, which was subsequently ligated to the pPG-PPT vector, resulting in the formation of pPG-Co1-6aa-S1-LTB. These four recombinant plasmids were then introduced into competent *L. paracasei 27-2* cells through electroporation, resulting in the generation of four recombinant strains. Positive transformants were assessed for chloramphenicol resistance. The plasmids exhibit stable replication facilitated by the repC replication origin. Transcription of the fusion gene is driven by the HCE constitutive promoter, while the T7g10 enhancer enhances transcription efficiency. The fusion protein, containing the PgsA anchoring domain, is directed to the cell membrane for anchoring to the cell wall via the LPXTG motif, facilitating display of the recombinant protein on the bacterial surface. Efficient termination of transcription is ensured by the rrnBT1T2 terminator. Detailed information regarding the primers utilized is provided in Table 1.

### 2.3. Protein Expression Analysis

#### 2.3.1. Western Blotting

A 10 mL MRS liquid medium with 10 μg/mL chloramphenicol was used to inoculate recombinant *Lactobacillus*, which was then incubated overnight. The bacterial culture was then centrifuged (12,000 rpm, 1 min), after which the supernatant was discarded. Following complete cell lysis with lysozyme, the resulting lysate was centrifuged, and the pellet was washed with sterile phosphate-buffered saline (PBS). Protein quantification utilized the bovine serum albumin (BSA) method, with 30 μg of protein loaded per well for Western blot analysis. Subsequently, the samples were transferred to PVDF membranes after SDS-PAGE resolution. For immunoblotting detection, the primary antibody used was a lab-prepared monoclonal mouse anti-S1 protein antibody (1:1000), and subsequently, we employed goat anti-mouse IgG (1:5000, Sigma, Ronkonkoma, NY, USA) conjugated with HRP. An ECL reagent (Thermo Scientific, Durham, NC, USA) was used to visualize immunoblots.

#### 2.3.2. Immunofluorescence Assay and Immunoelectron Microscopy

IFA was employed to verify the expression of target protein in recombinant *Lactobacillus*. Briefly, bacterial cultures were harvested, rinsed, and resuspended in PBS. The pellets were incubated with anti-S1 protein antibody (1:1000), followed by FITC-conjugated goat anti-mouse IgG (1:5000, Invitrogen, Carlsbad, CA, USA) in the dark. After DAPI (Invitrogen, Carlsbad, CA, USA) staining, samples were mounted on slides and observed under a fluorescence microscope (Zeiss, Oberkochen, Germany).

Colloidal gold nanoparticles were prepared by the sodium citrate reduction method [31], adjusted to pH 7.5 with 0.2 mol/L K_2_CO_3_, and filtered (0.22 μm). HRP-conjugated secondary antibody (Sigma, Ronkonkoma, NY, USA)was optimized by serial dilution, and the optimal concentration was conjugated with colloidal gold in the presence of 0.25% BSA for 30 min. The conjugates were washed by centrifugation (12,000 rpm, 4 °C), resuspended in PBS, and adjusted to an absorbance of 1.5 at 540 nm, then stored at 4 °C.

To further ascertain whether the target protein is expressed in a form that is anchored to the cell surface of recombinant *Lactobacillus*. Centrifugation, washing, permeabilization with 0.1% Triton X-100, and blocking with 3% BSA were all performed on recombinant *Lactobacillus*. Mouse anti-Flag antibody (1:1000, 4 °C, overnight) (Abmart, Shanghai, China) and gold-labeled secondary antibody (1:200, 37 °C, 1 h) were used to incubate the cells.

### 2.4. GM1-Binding Activity Assay and Biological Characterization of Recombinant Lactobacillus

To verify the functional activity of LTB, protein samples derived from recombinant *Lactobacillus* pPG-S1-LTB/*27-2* and pPG-Co1-6aa-S1-LTB/*27-2* were prepared at varying dilutions. The GM1-binding activity of the target protein was evaluated using an enzyme-linked immunosorbent assay (ELISA) (Wuhan Enzyme Immunoassay Biotechnology Co., Ltd., Wuhan, China). Microplates were coated overnight at 4 °C with 5 μg/mL bovine GM1 ganglioside dissolved in a carbonate–bicarbonate buffer (pH 9.6) and subsequently subjected to three washes with phosphate-buffered saline containing 0.05% Tween 20 (PBST). The coated plates were then blocked with 5% skimmed milk powder in PBS at 37 °C for 2 h. Protein samples processed at different concentrations (100-fold dilution, 10-fold dilution, stock solution, 10-fold concentration, and 30-fold concentration) were added in triplicate and incubated at 37 °C for 2 h, with blank buffer wells serving as negative controls. After washing with PBST, the plates were sequentially incubated with a 1:2000 dilution of anti-LTB monoclonal antibody at 37 °C for 1.5 h, followed by a 1:5000 dilution of horseradish peroxidase (HRP)-conjugated sheep anti-mouse IgG secondary antibody at 37 °C for 1 h, with PBST washes performed between each antibody application. To facilitate color development, a 3,3’,5,5’-tetramethylbenzidine (TMB) substrate solution was introduced and incubated at 37 °C in the absence of light for a duration of 15 to 20 min. The enzymatic reaction was subsequently halted by the addition of an ELISA stop solution, and the absorbance at 450 nm (A_450_) was quantified using a microplate reader. The GM1-binding activity was assessed based on the A_450_ values, with higher absorbance readings indicative of increased binding activity.

The growth kinetics of *L. paracasei 27-2* and recombinant *Lactobacillus* pPG-Co1-S1/*27-2*, pPG-Co1-6aa-S1/*27-2*, pPG-S1-LTB/*27-2*, and pPG-Co1-6aa-S1-LTB/*27-2* were systematically evaluated. To assess the stable inheritance of the exogenous gene and protein during the subculturing of recombinant *Lactobacillus*, the strains underwent 40 consecutive passages, with Western blot analysis conducted at every 10th generation., as well as tolerance to acidic conditions and bile salts.

### 2.5. Examination of Recombinant Lactobacillus’s Capacity to Target MoDCs

#### 2.5.1. Field-Emission Scanning Electron Microscope

MoDCs were isolated as described previously [32]. To produce immature DCs, DCs were cultivated for six days using granulocyte–macrophage colony-stimulating factor (GM-CSF; 20 ng/mL) (MCE, Monmouth Junction, NJ, USA) and interleukin-4 (IL-4; 20 ng/mL) (MCE, Monmouth Junction, NJ, USA). The morphology of MoDCs and recombinant *Lactobacillus* attached to MoDCs was examined by SEM (Hitachi, Tokyo, Japan) as described earlier [30]. Immature porcine MoDCs were incubated with pPG-6aa-S1/*27-2*, pPG-Co1-6aa-S1/*27-2*, pPG-Co1-6aa-S1-LTB/*27-2* and pPG-PPT/*27-2* for 2 h. and three PBS washes. The samples were then processed using field-emission [30,33].

#### 2.5.2. Flow Cytometry

Following a 30 min incubation period at 4 °C, the recombinant *Lactobacillus* was combined with carboxyfluorescein succinimidyl ester (CFSE) (MCE, Monmouth Junction, NJ, USA) and three PBS washes. Subsequently, recombinant *Lactobacillus* was combined with immature MoDCs and incubated (37 °C, 30 min). Flow cytometry was used to analyze the cells, while untreated immature MoDCs served as negative controls.

#### 2.5.3. Flat Colony Counting Method

After incubation with recombinant *Lactobacillus* at 37 °C for 30 min, MoDCs were washed with PBS. Following centrifugation, the bacterial pellet was plated on MRS agar. Each recombinant *Lactobacillus* group was assayed in triplicate, and identification was performed via PCR and colony counting.

### 2.6. Evaluation of Recombinant Lactobacillus’s Capacity to Target M Cells

#### 2.6.1. Immunofluorescence Staining on Tissue Sections

To evaluate the M cell-targeting capacity of pPG-Co1-S1/*27-2*, pPG-Co1-6aa-S1/*27-2*, pPG-Co1-6aa-S1-LTB/*27-2*, and pPG-PPT/*27-2*, the closed ileal loop and immunohistochemistry experiments were performed as previously described, with slight modifications [34,35]. In brief, the mice were euthanized, and closed ileal loops were established. One hundred microliters of pPG-Co1-S1/*27-2*, pPG-Co1-6aa-S1/*27-2*, pPG-Co1-6aa-S1-LTB/*27-2*, and pPG-PPT/*27-2* were administered into the ileal loop. Following incubation, the ileal loops were washed with PBS, fixed, and cryosectioned. The sample sections were stained with mouse anti-Flag antibody and goat anti-mouse IgG H&L (TRITC) (Invitrogen, Carlsbad, CA, USA). Rabbit anti-Gp2 antibody (Abmart, Shanghai, China) and goat anti-rabbit IgG H&L (FITC) were used to identify M cells in the Peyer’s patches, and nuclei were labeled with DAPI.

#### 2.6.2. Flat Colony Counting Method

Using sterile forceps and scissors, Peyer’s patches were meticulously excised from the murine intestinal wall, with strict avoidance of contamination by adjacent intestinal mucosa or adipose tissue. The excised patches were washed three times with PBS to eliminate surface contaminants, then transferred to sterile centrifuge tubes. Next, 1 mL of ice-cold sterile PBS was added, and the tissue was homogenized into a slurry. The homogenate was centrifuged (1500 rpm, 5 min, 4 °C) to collect the pellet. Subsequently, the pellet was spread onto MRS agar plates using a spreading rod and incubated overnight at 37 °C.

### 2.7. Animal Immunization and Sample Collection

The study utilized sixty-eight SPF BALB/c mice, comprising 17 males and 51 females, aged 8 to 9 weeks. And the mice were procured from Liaoning Changsheng Biotechnology Co., Ltd. (Shenyang, China). These mice were randomly assigned to seventeen groups, each consisting of one male and three females. Following isoflurane (RWD) anesthesia, the mice were euthanized by cervical dislocation method.

The recombinant *Lactobacillus* were grown for 12 h. The final concentration of 10^10^ CFU/mL was achieved by resuspending these strains in PBS. Oral administration of different formulations was given to groups of immunized mice (12 animals per group), with a PBS group serving as control (8 animals per group). Detailed information regarding the primers utilized is provided in Table 2. The immunization program ensured that the female mice would give birth subsequent to the completion of the second immunization. Samples were collected at various intervals following vaccination. The immune procedure of BALB/c mice is shown in Figure 1B.

Mice in each experimental group had serum samples taken on days 0, 7, 14, 21, and 28 after vaccination. The serum was subjected to centrifugation and stored at −40 °C for future analysis. Similarly, intestinal mucus and fecal samples were preserved at −40 °C until the quantification of their SIgA levels was conducted utilizing ELISA (Wuhan Enzyme Immunoassay Biotechnology Co., Ltd., Wuhan, China). Fecal pellets were suspended in PBS with phenylmethylsulphonyl fluoride and BSA, and incubated at 4 °C [36]. Pregnant mice gave birth after the secondary immunization. Intestinal mucus and serum from newborn mice were collected on days 1, 2 and 3, and SIgA and IgG antibodies specific to PEDV were measured using ELISA. During the data analysis phase, to mitigate the influence of subjective bias, experimenters and animal operators are kept unaware of which group of mice has been immunized with a specific strain of *Lactobacillus* or with PBS. In all experimental groups, all animals, experimental units, and data points were fully included without any exclusions, ensuring data integrity and a comprehensive analysis.

### 2.8. ELISA Analysis of Antibody Levels

ELISA was employed to quantify SIgA in intestinal mucus and feces of pregnant and newborn mice, as well as anti-PEDV IgG in the blood of newborn and immunized mice at specific time points. After two hours of blocking, diluted samples were incubated (1 h, 37 °C). Following a wash, the plates were incubated for one hour at 37 °C with goat anti-mouse IgG conjugated with HRP. OD_450nm_ was measured using a microplate reader.

### 2.9. PEDV Neutralization Assays

Serum and intestinal mucus samples were taken on the seventh day after immunization, then centrifuged and filtered to retrieve the supernatant. A two-fold serial dilution of the samples to a final dilution of 1:256 and combined with PEDV solution containing 100 TCID_50_. 2 × 10^5^ Vero cells were seeded into each well of a 96-well plate and cultured overnight at 37 °C in a 5% CO_2_ atmosphere until the cells attained 80–90% confluency. A Vero cell monolayer was treated with this mixture and incubated for two hours. The cell culture media were changed after the incubation period, and the supernatant was disposed of. After that, the mixture was kept at 37 °C with 5% CO_2_ for incubation. Observations of the cytopathic effect (CPE) were conducted on a daily basis. The Reed–Muench statistical approach was used to calculate the results [28].

### 2.10. Statistical Analysis

The data are expressed as the mean ± standard error of three replicates and were analyzed using GraphPad Prism version 10.1.2. To assess the significance between the treatment and control groups, Tukey’s multiple comparison test was performed after a one-way analysis of variance (ANOVA). A *p*-value below 0.05 was considered statistically significant.

## 3. Results

### 3.1. Development of Recombinant Lactobacillus and Identification of Recombinant Proteins

**Figure 2:**
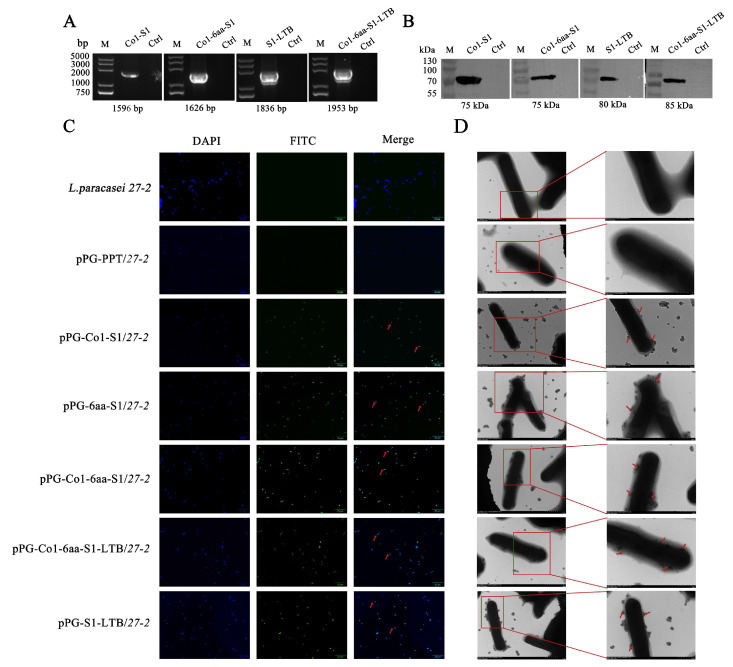
Identification and protein expression analysis of recombinant *Lactobacillus*. (**A**) PCR identification of recombinant plasmids. M: Trans 2k plus DNA marker. Western blot analysis (**B**); (**C**) IFA identification were used to confirm the fusion protein’s expression. (**D**) IEM identification were used to confirm the fusion protein’s expression. Red arrows denote the precise subcellular localization of the expressed proteins, as reported in the present study.

The construction results of the recombinant *Lactobacillus* are shown in Figure 2. The PCR results show that the target gene has been successfully constructed (Figure 2A). Protein production was analyzed by Western blotting (Figure 2B). The immunofluorescence assay was also used to verify that the target protein was expressed in recombinant *Lactobacillus* (Figure 2C). Transmission electron microscopy demonstrated that colloidal gold particles were distinctly distributed around the recombinant *Lactobacillus*. In contrast, no particle accumulation was detected on the surface of pPG-PPT/*27-2* (Figure 2D). According to these findings, the recombinant *Lactobacillus* surface successfully expressed the target proteins.

### 3.2. Biological Characterization Analysis

**Figure 3:**
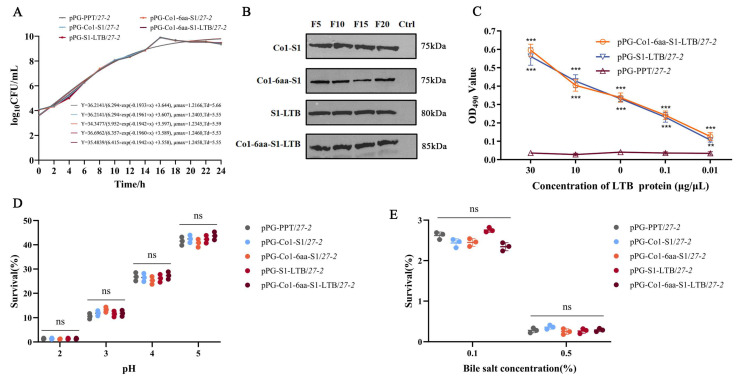
Biological characterization analysis. (**A**) Growth curve determination. (**B**) The protein stability of the recombinant *Lactobacillus* was assessed through Western blot analysis. (**C**) Analysis of binding activity of recombinant *Lactobacillus* expressing LTB protein. (**D**) Recombinant *Lactobacillus* under culture conditions with different pH. (**E**) Recombinant *Lactobacillus* under culture conditions with different concentrations of bile salt. Note: ** 0.01 < *p* < 0.05, *** *p* < 0.01, ns *p* > 0.05.

According to the data, the four recombinant *Lactobacillus* strains reached the stationary phase after about 16 h at 37 °C under static conditions, having begun the logarithmic growth phase about 4 h after inoculation. Furthermore, the growth characteristics of the recombinant *Lactobacillus* did not exhibit significant deviations from those of the wild-type strains (Figure 3A).

Western blotting investigations were carried out at intervals of every five passages to assess the strain’s genetic stability and the consistency of protein expression. All recombinant *Lactobacillus* demonstrated stable inheritance and consistent expression of the exogenous proteins (Figure 3B).

The binding activity of LTB and GM1 expressed by recombinant *Lactobacillus* was evaluated using the GM1-ELISA method (Figure 3C). The findings demonstrated that LTB expressed by recombinant *Lactobacillus* exhibited specific binding affinity to GM1, which was significantly different from the non-carrier bacterium pPG-PPT/*27-2*, which did not express LTB, across various concentrations (*p* < 0.01). Moreover, the binding efficacy increased with increasing concentration. Both recombinant *Lactobacillus* and wild-type strains exhibited specific tolerances to acid and bile salts (Figure 3D,E), without any statistically significant differences observed among them (*p* > 0.05).

### 3.3. Evaluation of Recombinant Lactobacillus’s Capacity to Target MoDCs

**Figure 4:**
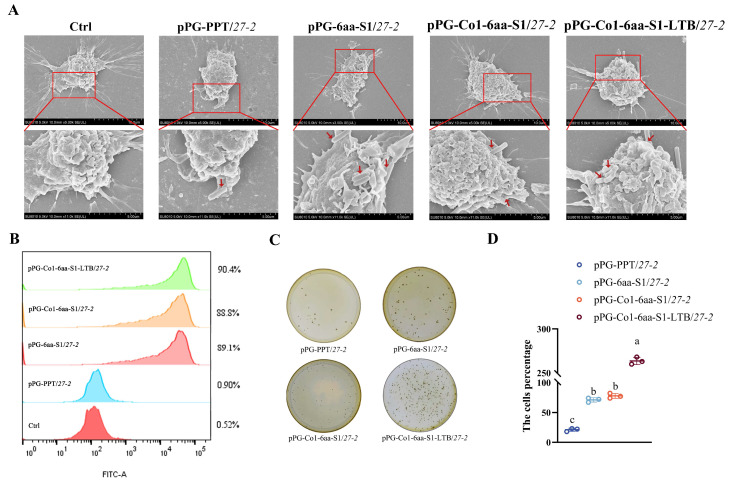
MoDCs’ targeting capacities and antibody-specific analysis for recombinant *Lactobacillus*. (**A**) Scanning electron micrographs show that swine MoDCs were able to identify and trap the recombinant *Lactobacillus*. The red arrow marks the exact location where the recombinant *Lactobacillus* developed in this study was internalized by dendritic cells. (**B**) Results of flow cytometry showing that MoDCs identified and collected recombinant *Lactobacillus*. (**C**,**D**) Data regarding the quantity of recombinant *Lactobacillus* colonies that were collected by MoDCs. Note: Different letters (a, b, c) indicate significant differences among groups at *p* < 0.05, and the same letter indicates no significant difference.

The pPG-6aa-S1/*27-2*, pPG-Co1-6aa-S1/*27-2*, and pPG-Co1-6aa-S1-LTB/*27-2* were effectively captured and identified by porcine MoDCs, whereas pPG-PPT/*27-2* failed to be captured by these cells (Figure 4A). Flow cytometric analysis revealed that the number of porcine MoDC-captured recombinant *Lactobacillus* strains, including pPG-6aa-S1/*27-2*, pPG-Co1-6aa-S1/*27-2*, and pPG-Co1-6aa-S1-LTB/*27-2*, was noticeably greater than that of pPG-PPT/*27-2* groups (*p* < 0.01) (Figure 4B). The flow cytometry results and the flat colony counting results were in agreement. (Figure 4C,D). These findings suggest that CTLA4-6aa could improve the recognition and capture of recombinant *Lactobacillus* by porcine MoDCs.

### 3.4. Evaluation of Recombinant Lactobacillus’s Capacity to Target M Cells

**Figure 5:**
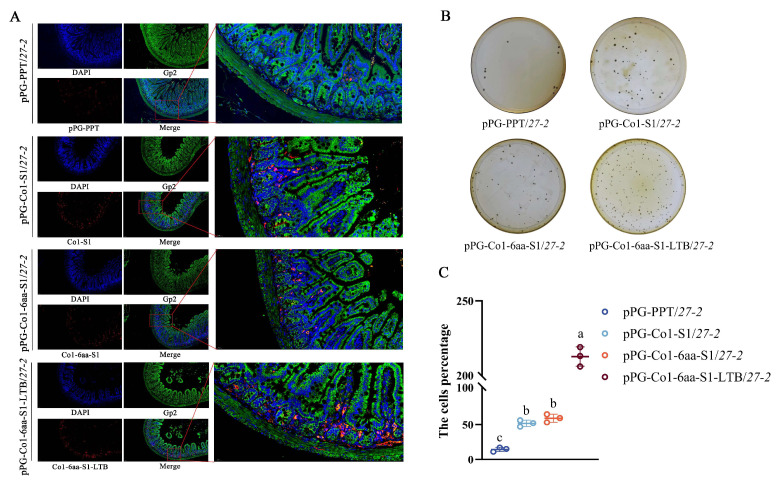
M cell-targeting abilities and antibody-specific analysis for recombinant *Lactobacillus*. (**A**) The recombinant *Lactobacillus* were injected into the closed ileal loop, which was then excised, washed, fixed, and frozen for sectioning after 1 h incubation. (**B**,**C**) Statistics on the number of colonies of *Lactobacillus* captured by M cells. Note: Different letters (a, b, c) indicate significant differences among groups at *p* < 0.05, and the same letter indicates no significant difference.

Immunofluorescence staining was conducted to assess the M cell-targeting efficacy of the recombinant *Lactobacillus*. The findings revealed that mice administered with pPG-Co1-S1/*27-2*, pPG-Co1-6aa-S1/*27-2*, or pPG-Co1-6aa-S1-LTB/*27-2* exhibited significantly more intense yellow fluorescence in the Peyer’s patches compared to those injected with pPG-PPT/*27-2*. This suggests that the M cell-targeting peptide Co1 enhances the targeting capability of recombinant *Lactobacillus* towards M cells (Figure 5A). The flat colony counting results aligned with the immunofluorescence results (Figure 5B,C).

### 3.5. Immune Responses That Are Triggered in Pregnant Mice When the Recombinant Lactobacillus Are Administered Orally

**Figure 6:**
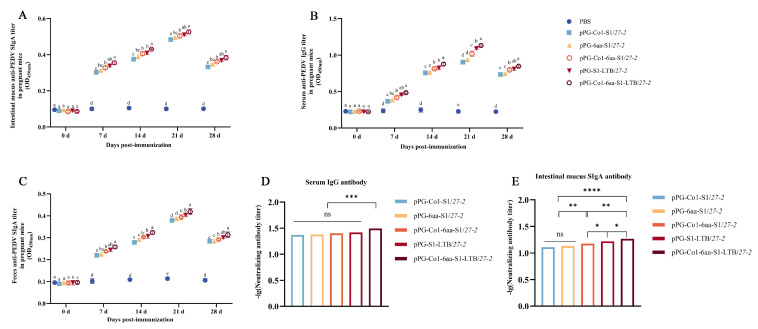
Anti-PEDV-specific IgG levels in the serum, SIgA levels in the intestinal mucus and feces of pregnant mice are listed in (**A**–**C**), respectively. (**D**,**E**) Measurement of neutralizing activity in intestinal serum IgG and mucus SIgA antibody levels in vaccinated pregnant mice. Note: Unique superscript letters (*p* < 0.05) indicate statistically significant differences between groups, while identical superscript letters (*p* > 0.05) indicate non-significant differences between groups. * *p* < 0.05, ** 0.01 < *p* < 0.05, *** *p* < 0.01, **** *p* < 0.001.

The quantity of anti-PEDV IgG antibodies generated by various recombinant *Lactobacillus* in pregnant mice was assessed by ELISA (Figure 6B). Following oral administration of the recombinant *Lactobacillus*, there was a progressive increase in antibody levels across all treatment groups. This increase was significantly greater compared to the levels observed in the PBS group. Notably, the oral administration of recombinant *Lactobacillus* pPG-Co1-6aa-S1/*27-2*, which incorporates both DC-targeting and MC-targeting peptides, caused a notable significant elevation in secretory IgG antibody levels compared to the recombinant *Lactobacillus* pPG-Co1-S1/*27-2* and pPG-6aa-S1/*27-2*, which contain single-targeting peptides (*p* < 0.05). Moreover, the oral administration of recombinant *Lactobacillus* pPG-Co1-6aa-S1-LTB/*27-2*, which incorporates dual-targeting peptides and adjuvants, resulted in significantly elevated IgG antibody levels compared to all other groups (*p* < 0.05). The ELISA method was used to measure the amounts of SIgA antibodies in the intestinal and fecal samples of pregnant mice were measured by ELISA (Figure 6A,C). The results suggest that recombinant *Lactobacillus* can effectively trigger mucosal immune responses in pregnant mice. Moreover, the immunogenic efficacy of the recombinant *Lactobacillus* pPG-Co1-6aa-S1-LTB/*27-2* surpasses that of the other groups evaluated.

### 3.6. Determination of Neutralizing Antibody Activity in Orally Immunized Pregnant Mice

The research employed the Reed–Muench two-component method to evaluate the neutralizing effect of IgG (Figure 6D) and SIgA (Figure 6E) against PEDV. These findings showed that all recombinant *Lactobacillus* producing S1 were able to successfully stimulate pregnant mice to produce antibodies specific to PEDV. Moreover, the group of recombinant *Lactobacillus* carrying the pPG-Co1-6aa-S1-LTB/*27-2* exhibited superior immune effects and higher antibody neutralizing activity.

### 3.7. Measurement of the Amount of Cytokines in Pregnant Mice’s Serum

**Figure 7:**
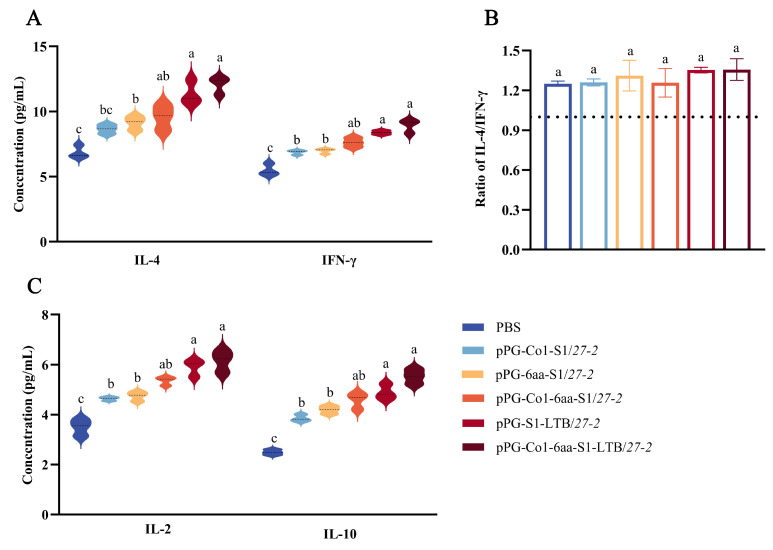
(**A**–**C**) Measurements of the blood cytokine levels in pregnant mice that have received recombinant *Lactobacillus* and PBS vaccinations. The mean ± SEM of three separate experiments is used to illustrate the results. Note: Unique superscript letters (*p* < 0.05) indicate statistically significant differences between groups, while identical superscript letters (*p* > 0.05) indicate non-significant differences between groups.

Serum samples were taken from pregnant mice that had received an oral recombinant *Lactobacillus* vaccination seven days after the initial inoculation. ELISA was utilized to quantify the cytokine expression levels. The results, depicted in Figure 7, indicated that the recombinant *Lactobacillus* induced significantly higher levels of IFN-γ, IL-2, IL-4 and IL-10 cytokines in serum compared to the control group. Importantly, the recombinant *Lactobacillus* pPG-Co1-6aa-S1-LTB/*27-2* showed a notable rise in cytokine levels compared to the other groups (*p* < 0.05). Overall, the results indicate that all recombinant *Lactobacillus* that produced S1 were successful in triggering cellular immune responses in pregnant mice of the Th1 and Th2 types.

### 3.8. Evaluation of Anti-PEDV Specific Antibodies Present in the Intestinal Mucus of Neonatal Mice

**Figure 8:**
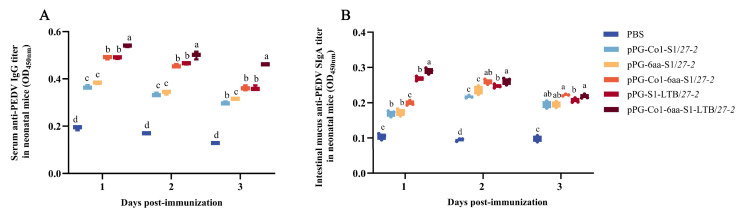
Levels of anti-PEDV specific antibodies of neonatal mice. Quantities of anti-PEDV specific IgG (**A**) and SIgA (**B**) antibodies in the intestinal mucus of neonatal mice. Note: Unique superscript letters (*p* < 0.05) indicate statistically significant differences between groups, while identical superscript letters (*p* > 0.05) indicate non-significant differences between groups.

Following the second vaccination, pregnant mice gave birth to neonatal mice. An ELISA kit was used to assess the serum’s level of anti-PEDV specific IgG antibody (Figure 8A). Intestinal mucus was collected from the newborn pups on days 1, 2, and 3, and the anti-PEDV specific SIgA antibody level present in the mucus was measured using an ELISA kit (Figure 8B). Over the course of several days, these antibodies progressively rose and were noticeably greater than the PBS group (*p* < 0.05). In comparison to recombinant *Lactobacillus* expressing single-target peptides, the oral administration of recombinant *Lactobacillus* pPG-Co1-6aa-S1/*27-2*, which incorporates both DC-targeting and MC-targeting peptides, led to a substantial elevation in antibody quantities. The SIgA antibody levels induced by pPG-S1-LTB/*27-2* with adjuvant were significantly higher than those induced by recombinant *Lactobacillus* fused with dual-targeting peptides (*p* < 0.05). Furthermore, oral administration of recombinant *Lactobacillus* with dual-targeting peptides and adjuvant, pPG-Co1-6aa-S1-LTB/*27-2*, resulted in significantly highest SIgA antibody levels. These findings indicated that all recombinant *Lactobacillus* that produce S1 may successfully induce mucosal immune responses in neonatal mice, and that maternal antibodies can be transferred from female mice to their offspring.

## 4. Discussion

Piglets of all age groups are susceptible to PED, an acute intestinal infectious disease that is globally prevalent [37]. Neonatal piglets are particularly susceptible, exhibiting high morbidity and mortality rates that hinder the progress of pig farming and cause substantial economic losses to the global swine industry. Therefore, creating protective vaccines for PEDV is still a top priority. PEDV presents challenges due to its rapid mutation rate and the difficulties associated with its prevention and control. Inactivated and live attenuated vaccines are currently the main types of vaccinations on the market. Inactivated vaccines offer benefits such as low production costs and high yield; however, they are also associated with drawbacks, including low immunogenicity [38]. Live attenuated vaccines elicit robust immune responses but pose a risk of reverting to a more virulent strain [39]. Therefore, there is a critical demand for vaccines that are both safe and effective, while also demonstrating strong immunogenicity.

The PEDV CH/HLJ/18 strain exhibits high pathogenicity in neonatal piglets and is classified within the GIIa genotype [26]. The S-trimer may be more appropriate than the RBD for subunit vaccines targeting alpha-CoVs [40]. The mRNA vaccine encoding the full PEDV spike (S) protein is more effective than the multi-epitope chimeric spike (Sm) protein mRNA vaccine in eliciting antibody and cellular immune responses in mice [41]. Epitopes for neutralizing antibodies are found in two separate regions of S1, which are responsible for binding to carbohydrate and protein molecules on the cell surface [42]. In this study, the S1 subunit, identified as the immunodominant region of the S protein, was chosen as the immunogen. The S1 gene sequence analyzed in this study was cloned from the CH/HLJ/18 strain, indicating that an immunogen based on this local isolate could potentially aid in the management and prevention of regional outbreaks. Therefore, the results of this research suggest that pPG-Co1-6aa-S1-LTB/*27-2* may serve as a novel mucosal vaccine to a certain extent. The primary challenge currently confronting us is how to elicit a robust, efficient, and durable immune response to *lactic acid bacteria* expressing the desired antigen. Consequently, this study involves the construction of recombinant *Lactobacillus* fused with various targeted peptides, immune adjuvants, and the PEDV protective antigen S1. The objective is to investigate whether the combined application of multiple mucosal immune-enhancing elements can significantly augment the body’s immune response.

Numerous prototype vaccines associated with *Lactobacillus* have been formulated. Ding Guojie [43] has engineered a recombinant *Lactobacillus* quadrivalent vaccine capable of continuously expressing the α, ε, β1 and β2 toxins of Clostridium perfringens, and has investigated its immunogenic properties in mice via oral administration. Concurrently, based on the CRISPR-Cas9 D10A system, a stable trophic-deficient *Lactobacillus △Alr HLJ-27* was constructed. By inserting the *VP4* gene of Porcine Rotavirus (PoRV) into the genome, the recombinant *Lactobacillus* effectively stimulate mucosal immune responses mediated by secretory SIgA, in addition to eliciting humoral immune responses facilitated by IgG [44]. Antigen uptake is facilitated through transcytosis across the M cell membrane and the extension of dendritic cell dendrites into the lumen. The core neutralizing epitope (COE) of the PEDV spike protein was included in an oral recombinant *Lactobacillus* vaccine that was created in some experiments using an MC-targeting peptide and a DC-targeting peptide. The vaccine specifically targets intestinal M cells and dendritic cells. This probiotic vaccine has demonstrated efficacy in inducing mucosal and IgG-mediated humoral immune responses following in vivo oral administration [28,45]. Moreover, targeting antigens to the surface receptors of DCs can enhance the immune response by augmenting the DCs’ capacity for antigen recognition and uptake. The LYPPPY (6aa) sequence found in the extracellular part of the porcine CTLA4 gene is essential for its interaction with porcine DCs. The fusion expression of the extracellular LYPPPY sequence of the CTLA4 gene can enhance the antigen recognition and uptake capabilities of porcine MoDCs, thereby improving the immune function of these cells [30]. When used in conjunction with other antigens for co-stimulation, the LTB functions as an effective mucosal immune adjuvant. Furthermore, the incorporation of targeted peptides and immunological adjuvants can further potentiate the immune response [46,47,48,49]. This study demonstrated significant differences in SIgA levels in feces, serum, and intestinal mucus from day 7 post-immunization compared to the PBS group. These findings suggest that targeting ligands and adjuvants can enhance immunogenicity more rapidly in the early stages, particularly with respect to SIgA levels, underscoring the potential efficacy of our oral vaccines.

Our findings indicate that recombinant *Lactobacillus* incorporating both DCs and M cell-targeting peptides are more effectively internalized at the mucosal surface, leading to enhanced SIgA and systemic IgG responses compared to strains with single-target or non-targeted peptides [35,50]. This evidence suggests that the dual-targeting strategy synergistically amplifies immune priming by promoting both antigen transcytosis and presentation. While previous research has shown that targeting either DCs or M cells individually can partially enhance mucosal responses, our study demonstrates that combining these strategies results in a more robust and sustained immune response. Therefore, this dual-targeting strategy provides a promising framework for developing oral vaccines against PEDV and other enteric pathogens.

This study demonstrated that the LTB expressed by the recombinant *Lactobacillus* pPG-Co1-6aa-S1-LTB/*27-2* and pPG-S1-LTB/*27-2* exhibited specific GM1-binding activity, which was significantly different from that of the non-carrier bacterium pPG-PPT/*27-2* lacking LTB, across various concentrations (*p* < 0.01). Furthermore, in comparison to pPG-Co1-6aa-S1/*27-2*, the recombinant *Lactobacillus* pPG-Co1-6aa-S1-LTB/*27-2* elicited higher levels of IgG and SIgA. These findings indicate that LTB possesses immunomodulatory properties, facilitating the induction of Th1 and Th2 responses. The evaluation also included neutralizing antibodies, which serve as qualitative indicators of vaccine effectiveness for multiple approved vaccines [51]. In the evaluation of PEDV-neutralizing activity, the Col-6aa-S1-LTB group showed the highest neutralization potential, indicating that pPG-Col-6aa-S1-LTB/*27-2* would be a good vaccine candidate to prevent PEDV infections.

While piglets are considered the gold-standard model for assessing PEDV pathogenesis and vaccine efficacy, the utilization of mice offers a practical and informative alternative during the initial phases of research. Mice present several advantages for evaluating the immunogenicity of candidate strains, including low maintenance costs, a well-characterized genetic background, and conserved mucosal immune features such as Peyer’s patches, M cells, and IgA responses [52,53]. Their short reproductive cycle and high breeding efficiency facilitate rapid longitudinal studies with large sample sizes, significantly reducing the screening cycle for recombinant strains, such as Lactobacillus expressing PEDV antigens, compared to porcine models [54,55]. Given the intestinal tropism of PEDV, the murine intestinal mucosal microenvironment, which includes gut microbiota composition and epithelial barrier function, exhibits a high degree of homology with that of pigs, thereby ensuring reliable extrapolation of immune response trends to the target host [56]. Moreover, standardized experimental protocols for mouse models, including oral gavage, sample collection, and immune detection, enhance the reproducibility and comparability of immunogenicity data, establishing a robust foundation for subsequent validation in piglets.

The transmission of PEDV to neonatal piglets can occur through milk, which acts as a conduit for the acquisition of maternal antibodies by the piglets [57]. Owing to the high virulence of PEDV and the immaturity of the neonatal piglet immune system, targeting maternal passive lactogenic immunity is the most effective method to protect neonatal piglets against PED [58]. This immunity, which is induced through the gut-mammary gland (MG)-SIgA axis during pregnancy and lactation, represents the most promising and effective strategy for protecting nursing piglets from PEDV-induced disease [59]. In mice, maternal antibodies are also passed to the offspring through the placenta and colostrum [60,61]. Due to the challenges associated with collecting milk from pregnant females, antibody levels were assessed by obtaining intestinal mucus from neonatal mice. Research conducted in murine models has shown that all five recombinant *Lactobacillus* can elicit robust humoral immune responses, both locally and systemically. Offspring of female mice immunized with recombinant *Lactobacillus* exhibited anti-PEDV specific SIgA antibodies in their intestinal mucus, with the antibody levels in the intestinal mucus of neonatal pups being assessed over a three-day period. Over time, the levels progressively increased, suggesting the acquisition of maternal antibodies. The antibodies elicited by these five recombinant *Lactobacillus* demonstrated neutralizing activity against PEDV, thereby suggesting their potential for protection against PEDV infection. The efficacy of these recombinant *Lactobacillus* in preventing and controlling PEDV in piglet herds requires further evaluation in future studies. This study demonstrated that the elevated levels of PEDV-specific SIgA in the intestinal mucus of neonatal mice, born to mother mice immunized with recombinant *Lactobacillus*, offer significant reference value and a theoretical foundation for research on the passive immune effects mediated by colostrum in piglets.

Further studies should focus on swine zoopery to investigate immune efficacy and protection. Although the BALB/c mouse is not a susceptible animal model for PEDV, our results to some extent indicate that surface-displaying pPG-Col-6aa-S1-LTB/*27-2* could serve as a novel mucosal vaccine that provides opportunities for PEDV vaccine development.

Th1 cells mainly generate IL-2 and IFN-γ, which are crucial for attacking intracellular pathogens and promoting IgG production to defend against viruses. In contrast, Th2 cells primarily release IL-4 and IL-10, which are crucial for stimulating B-cell proliferation and the production of antibodies, hence enhancing humoral immunity [62,63]. A comprehensive analysis of the secretion levels of IL-10, IL-4, IL-2, and IFN-γ was performed to ascertain the equilibrium between Th1 and Th2 immune responses [64,65]. The results demonstrated that recombinant *Lactobacillus* expressing S1 antigens significantly enhanced the secretion of these cytokines. Particularly, the group administered with recombinant *Lactobacillus* pPG-Co1-6aa-S1-LTB/*27-2* exhibited significantly higher cytokine levels compared to other groups. Additionally, the ratio of IL-4 to IFN-γ in the recombinant *Lactobacillus* group is higher than 1. Th2 cells enhance type 2 immunity by producing cytokines. Type 2 immunity constitutes a specialized immune response that forms a protective barrier on mucosal surfaces, aiding in the elimination of pathogens through the coordinated action of innate and adaptive immune mechanisms [65].

This study effectively engineered *Lactobacillus paracasei 27-2* to augment mucosal immunity and express the protective antigen S1 of PEDV. The recombinant *Lactobacillus* pPG-Co1-6aa-S1-LTB/*27-2*, with dual-targeting capabilities and the immune adjuvant LTB, demonstrated a robust immune response in mice. These results imply that oral administration of recombinant *Lactobacillus* expressing S1 antigens successfully stimulated Th1 and Th2 cell immunological responses in mice, promoting humoral, mucosal, and cellular immunity all at once. The immunogenic properties of this recombinant *Lactobacillus* were initially assessed using a pregnant mouse model, thereby establishing a basis for subsequent animal experiments in piglets.

## 5. Conclusions

In summary, this study developed a recombinant *Lactobacillus paracasei* that expresses a multicomponent fusion antigen. This antigen comprises the PEDV S1 protein, peptides targeting M cells and dendritic cells, and the mucosal adjuvant LTB, positioning it as a candidate for an oral vaccine. Oral immunization of pregnant mice with this strain elicited robust PEDV-specific humoral immunity, characterized by serum IgG and intestinal/fecal SIgA with neutralizing activity, as well as cellular immunity, indicated by elevated levels of IFN-γ, IL-2, IL-4, and IL-10. The resulting oral vaccine effectively induces robust mucosal immunity, humoral immunity, and cellular immune responses, thereby offering maternal immune protection. This approach presents a practical and promising strategy for the prevention and control of PEDV, with the potential to reduce the economic losses caused by PEDV in the pig farming industry.

## Figures and Tables

**Table 1:** Details of primers used in this study.

Primers	Primer Sequence (5′–3′)	Target	Amplicon Length (bp)
S1-F1	GAGCTCATGTGCATTGGTTATGCTGCCAATGTATT	S1	1509
S1-R1	CCAGGACCATACGTAGTAAAAGAAACCAGGCAACT		
Co1-F	GAGCTCATGTCTTTTCATCAATTACCAGCTAGATCTCCATTACCT***TACGTATGGTCC***TGGTCCTGCATTGGTTATGCTGCC	Co1-S1	1596
Co1-R	GGGCCCCTACTTATCGTC		
LTB-F	***TACGTATGGTCCTGGTCC*** GCTCCCCAGACTATTAC	LTB	327
LTB-R	GGGCCCCTActtatcgtcgtcatccttgtaaTCGTTTTTCATACTGATTGCC		
C6-F	GAGCTCATGTCTTTTCATCAATTACCAGCTAGATCTCCATTACCT***GGTGGCGGTGGCTCA*CTGTACCCACCACCCTAC*GAAGCCGCAGCCAAAGAG***	Co1-6aa-S1-LTB	1953

Note: The underlined segment denotes the introduced restriction site, while the bold segment represents the LYPPPY peptide sequence derived from porcine CTLA4, which serves as the dendritic cell-targeting peptide (6aa) [30]. The sequence in lowercase corresponds to the Flag tag. The bold italicized portion signifies the rigid linker sequence, and the double-underlined segment identifies the M cell-targeting peptide.

**Table 2:** Grouping of experimental mice.

Groups	Strain	Oral Dose	Number of Mice (pcs)	Oral Procedure
I	pPG-Co1-S1/*27-2*	200 μL	12	Immunization for three consecutive days, at two weeks apart, for a total of two immunizations.
II	pPG-6aa-S1/*27-2*		12	
III	pPG-Co1-6aa-S1/*27-2*		12	
IV	pPG-Co1-6aa-S1-LTB/*27-2*		12	
V	pPG-S1-LTB/*27-2*		12	
Control	PBS		8	

## Data Availability

The data that support the findings of this study are openly available in Mendeley Data at http://doi.org/10.17632/rdfc9hb88h.1.

## Abbreviations

The following abbreviations are used in this manuscript:

PED	Porcine Epidemic Diarrhea
PEDV	Porcine Epidemic Diarrhea Virus
SIgA	Secretory immunoglobulin A
IgG	Immunoglobulin G
LTB	Heat-labile enterotoxin B subunit
DCs	Dendritic cells
MoDCs	Monocyte-derived dendritic cells
IFN-γ	Interferon-γ
IL-2	Interleukin-2
IL-10	Interleukin-10
MRS	Man Rogosa and Sharpe
PBS	Phosphate-buffered saline
SDS	Sodium dodecyl sulfate
PVDF	Polyvinylidene fluoride
HRP	Horseradish peroxidase
FITC	Fluorescein isothiocyanate
CFSE	Carboxyfluorescein succinimidyl ester
TRITC	Tetramethylrhodamine isothiocyanate
CPE	Cytopathic effect
ELISA	Enzyme-linked immunosorbent assay
GM-CSF	Granulocyte–macrophage colony-stimulating factor
LAB	Lactic acid bacteria
IL-4	Interleukin-4
